# ^1^H-NMR and ^13^C-NMR dataset for some oxidative metabolites of CRA13 and their analogs

**DOI:** 10.1016/j.dib.2018.09.069

**Published:** 2018-10-10

**Authors:** Ahmed H.E. Hassan, Min Chang Cho, Hye In Kim, Ji Seul Yang, Kyung Tae Park, Yong Sup Lee

**Affiliations:** a Medicinal Chemistry Laboratory, Department of Pharmacy, College of Pharmacy, Kyung Hee University, Seoul 02447, Republic of Korea; b Department of Medicinal Chemistry, Faculty of Pharmacy, Mansoura University, Mansoura 35516, Egypt; c Department of Life and Nanopharmaceutical Sciences, Kyung Hee University, Seoul 02447, Republic of Korea; d KHU-KIST Department of Converging Science and Technology, Kyung Hee University, Seoul 02447, Republic of Korea

## Abstract

CRA13 (CB-13; SAB-378) is a dual CB_1_R/CB_2_R agonist cannabinoid agent developed by Novartis Pharma. Upon administration, it undergoes metabolism to oxidative metabolites. Herein, the ^1^H-NMR and ^13^C-NMR dataset of some oxidative metabolites and analogs thereof are presented for further analysis and comparison purposes, for whom may be interested.

**Specifications table**

**Table:** 

Subject area	Chemistry
More specific subject area	Structural characterization
Type of data	Figures
How data was acquired	Nuclear magnetic resonance (NMR) spectral analyses were performed using a Brucker Avance 400 spectrometer (400 MHz for ^1^H-NMR) or Agilent 500 spectrometer (125 MHz for ^13^C-NMR).
Data format	*Raw*
Experimental factors	Sample solutions were prepared with deuterated CDCl_3_ or CD_3_OD.
Experimental features	Detection temperature was set at 25 °C. Samples were scanned 16 times for ^1^H-NMR spectra measurement, and scanned 1 h for ^13^C-NMR measurement
Data source location	Kyung Hee University, Seoul, Republic of Korea
Data accessibility	Data is provided in the article
Related research article	A.H.E. Hassan, M.C. Cho, H.I. Kim, J.S. Yang, K.T. Park, J.Y. Hwang, C.G. Jang, K.D. Park, Y.S. Lee, Synthesis of oxidative metabolites of CRA13 and their analogs: Identification of CRA13 active metabolites and analogs thereof with selective CB_2_R affinity, *Bioorg. Med. Chem.***26**, 2018, 5069–5078, doi:10.1016/j.bmc.2018.09.007.

**Value of the data**

• CRA13 (CB-13; SAB-378) is a controlled cannabinoid substance in China and therefore a reference data for its metabolites are required.
• The presented data provides reference that might be useful for detection of metabolites of CRA13 and related cannabinoids in biological samples.
• In addition. It might be helpful in the assignment of signals of molecules containing di(naphthalen-1-yl)methanone moiety.
• Also, the shown splitting and chemical shifts is helpful to structural analysis of related cannabinoids.

## 1 Data

The data presented herein describe the acquired for ^1^H-NMR and ^13^C-NMR spectra of hydroxy and carboxy metabolites of CRA13, as well as, methyl ester and analogs of the hydroxy, carboxy and methyl ester [1], [2]. Thus, a total of eight compounds were chemically synthesized and NMR data of pure samples were acquired in deuterated chloroform or deuterated methanol. These data might be useful for detection of metabolites of these controlled cannabinoids compounds and for structural assignment of compounds possessing di(naphthalen-1-yl)methanone moiety. The data are presented as figures with enlargement of proton peaks to clarify their splitting pattern.

## 2 Experimental design, materials, and methods

The compounds were chemically synthesized and purified by column chromatography as described in [1]*.* The samples were dissolved in CDCl_3_ or CD_3_OD then NMR spectra were acquired using a Brucker Avance 400 spectrometer (400 MHz) for ^1^H-NMR or Agilent 500 spectrometer (125 MHz) for ^13^C-NMR at 25 °C. The NMR peaks of the acquired NMR spectra are shown in Fig. 1, Fig. 2, Fig. 3, Fig. 4, Fig. 5, Fig. 6, Fig. 7, Fig. 8, Fig. 9, Fig. 10, Fig. 11, Fig. 12, Fig. 13, Fig. 14, Fig. 15, Fig. 16.

**Fig. 1:**
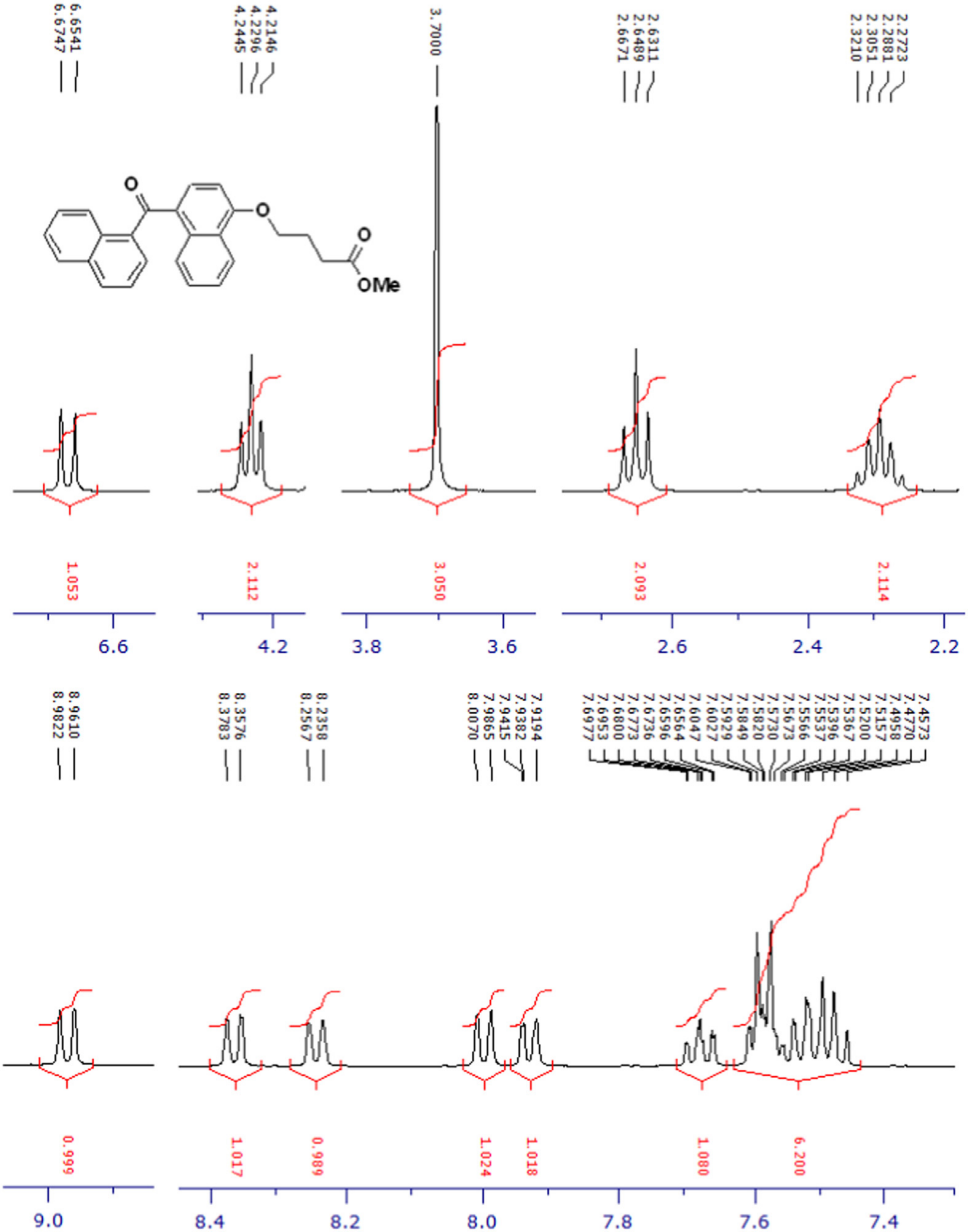
^1^H-NMR spectrum of four carbons alkyl chain analog of methyl ester of terminally oxidized carboxylic acid metabolite of CRA13 (CDCl_3_, 400 MHz).

**Fig. 2:**
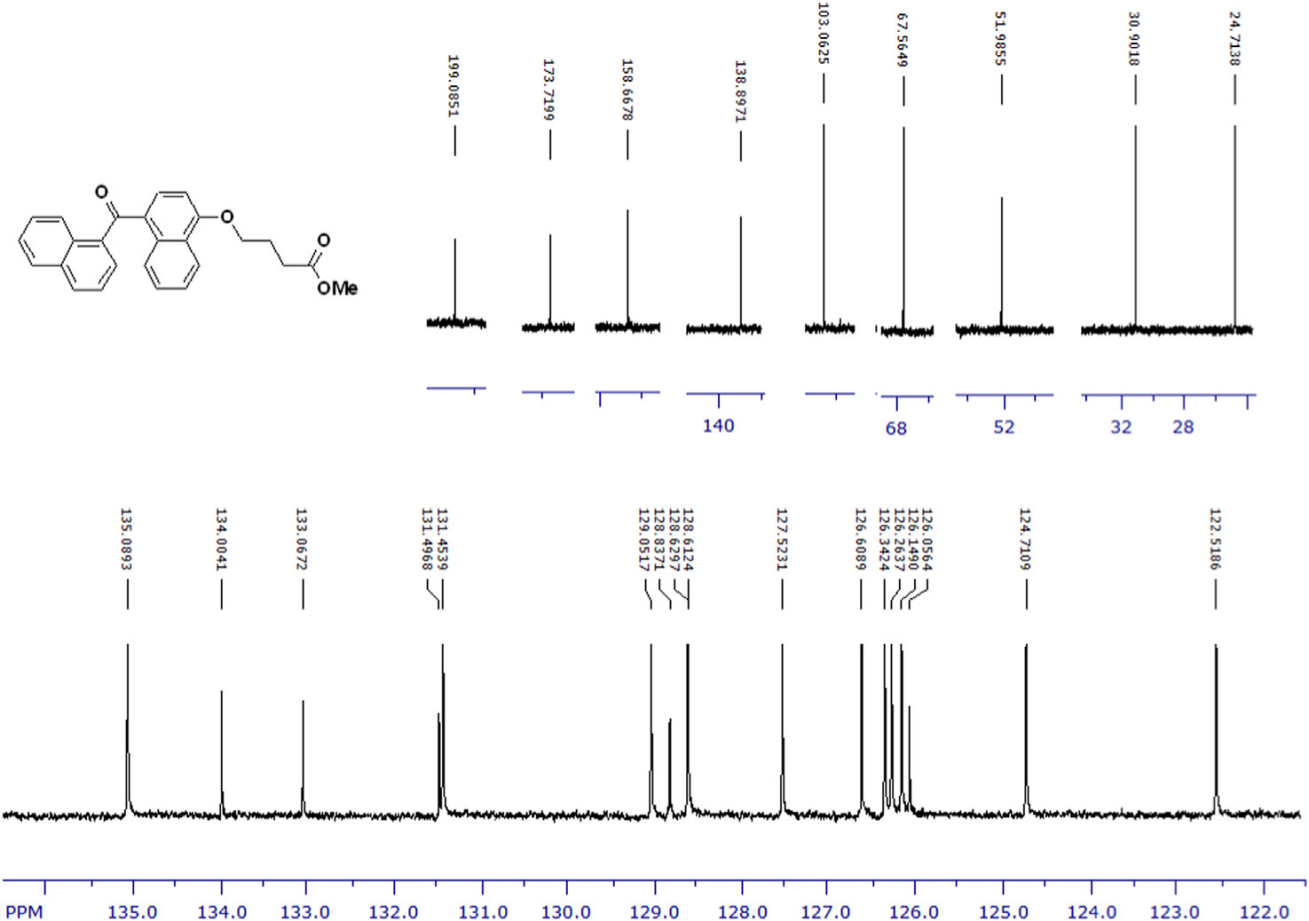
^13^C-NMR spectrum of four carbons alkyl chain analog of methyl ester of terminally oxidized carboxylic acid metabolite of CRA13 (CDCl_3_, 125 MHz).

**Fig. 3:**
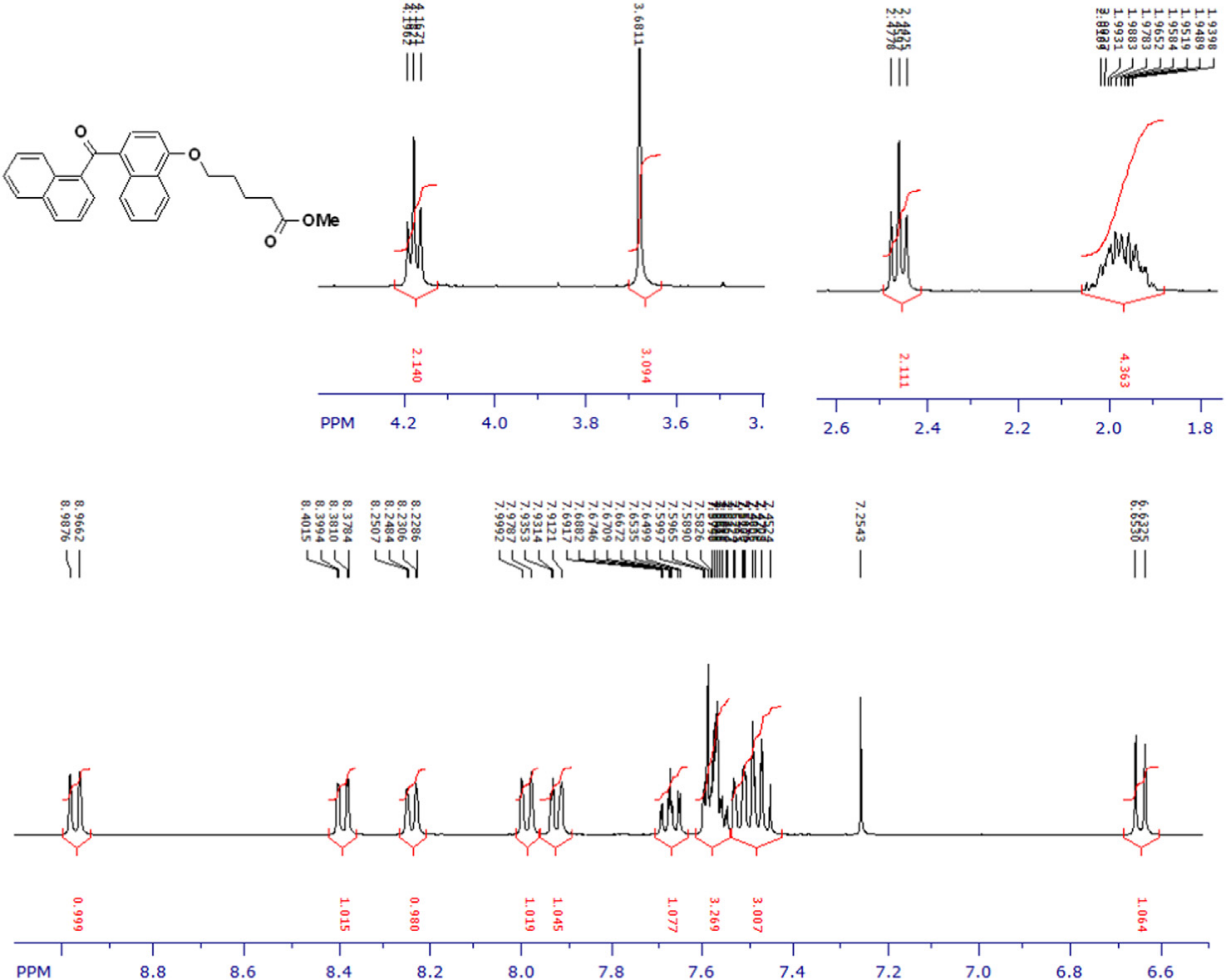
^1^H-NMR spectrum of methyl ester of terminally oxidized carboxylic acid metabolite of CRA13 (CDCl_3_, 400 MHz).

**Fig. 4:**
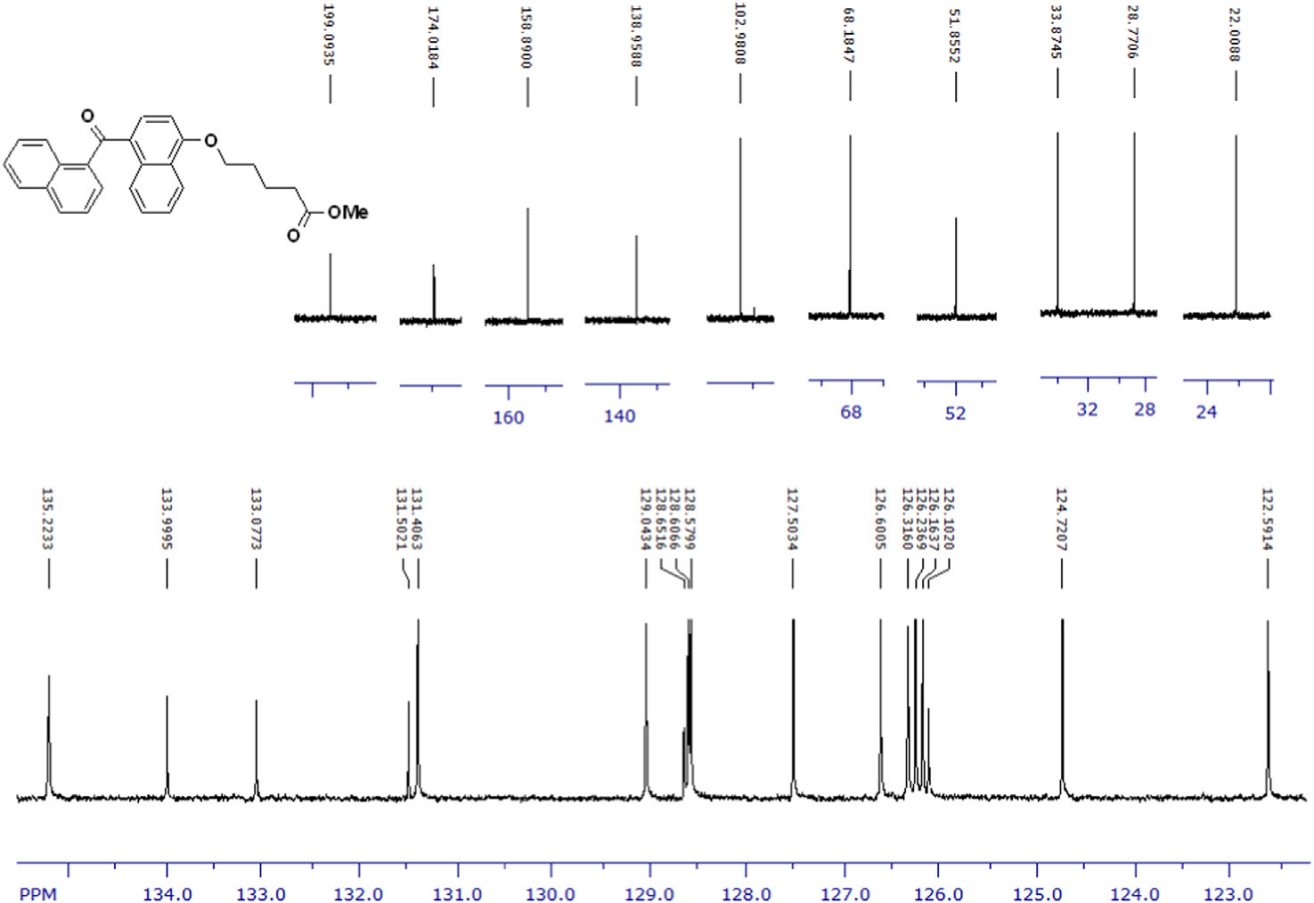
^13^C-NMR spectrum of methyl ester of terminally oxidized carboxylic acid metabolite of CRA13 (CDCl_3_, 125 MHz).

**Fig. 5:**
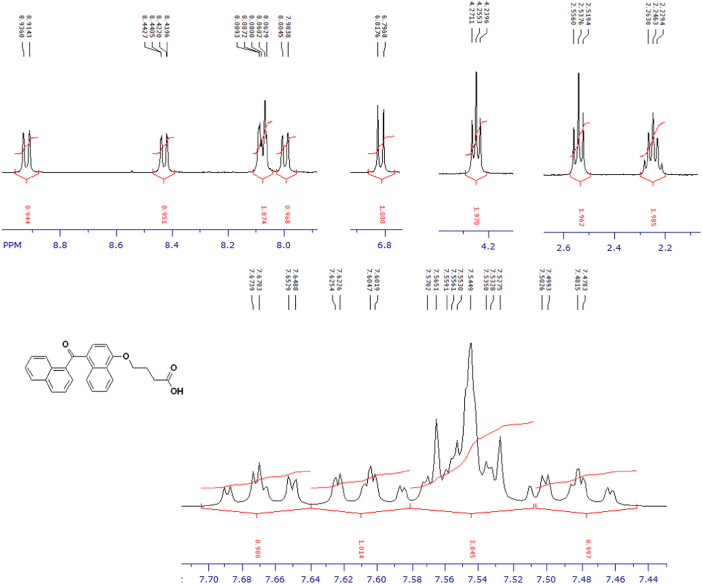
^1^H-NMR spectrum of four carbons alkyl chain analog of terminally oxidized carboxylic acid metabolite of CRA13 (CD_3_OD, 400 MHz).

**Fig. 6:**
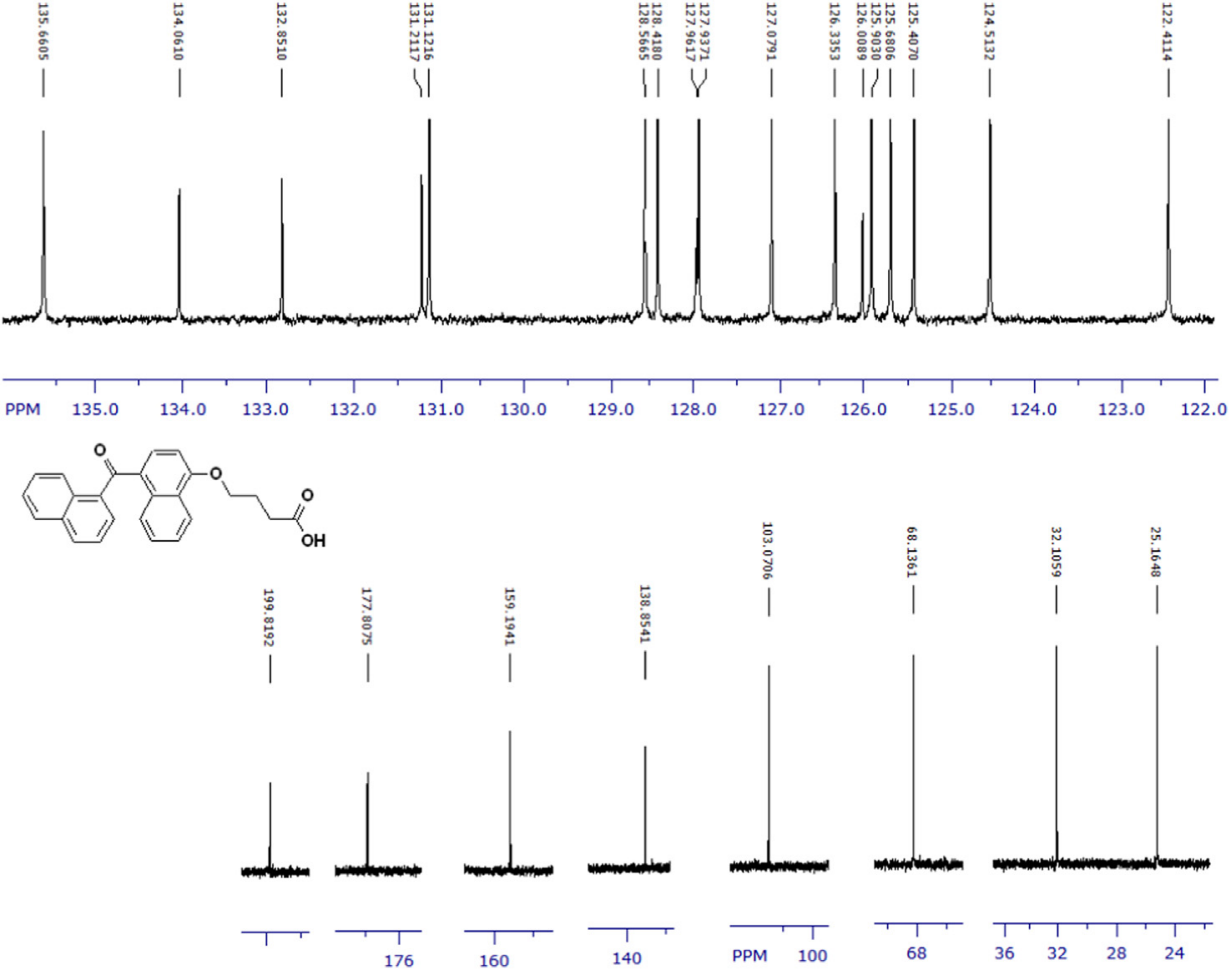
^13^C-NMR spectrum of four carbons alkyl chain analog of terminally oxidized carboxylic acid metabolite of CRA13 (CD_3_OD, 125 MHz).

**Fig. 7:**
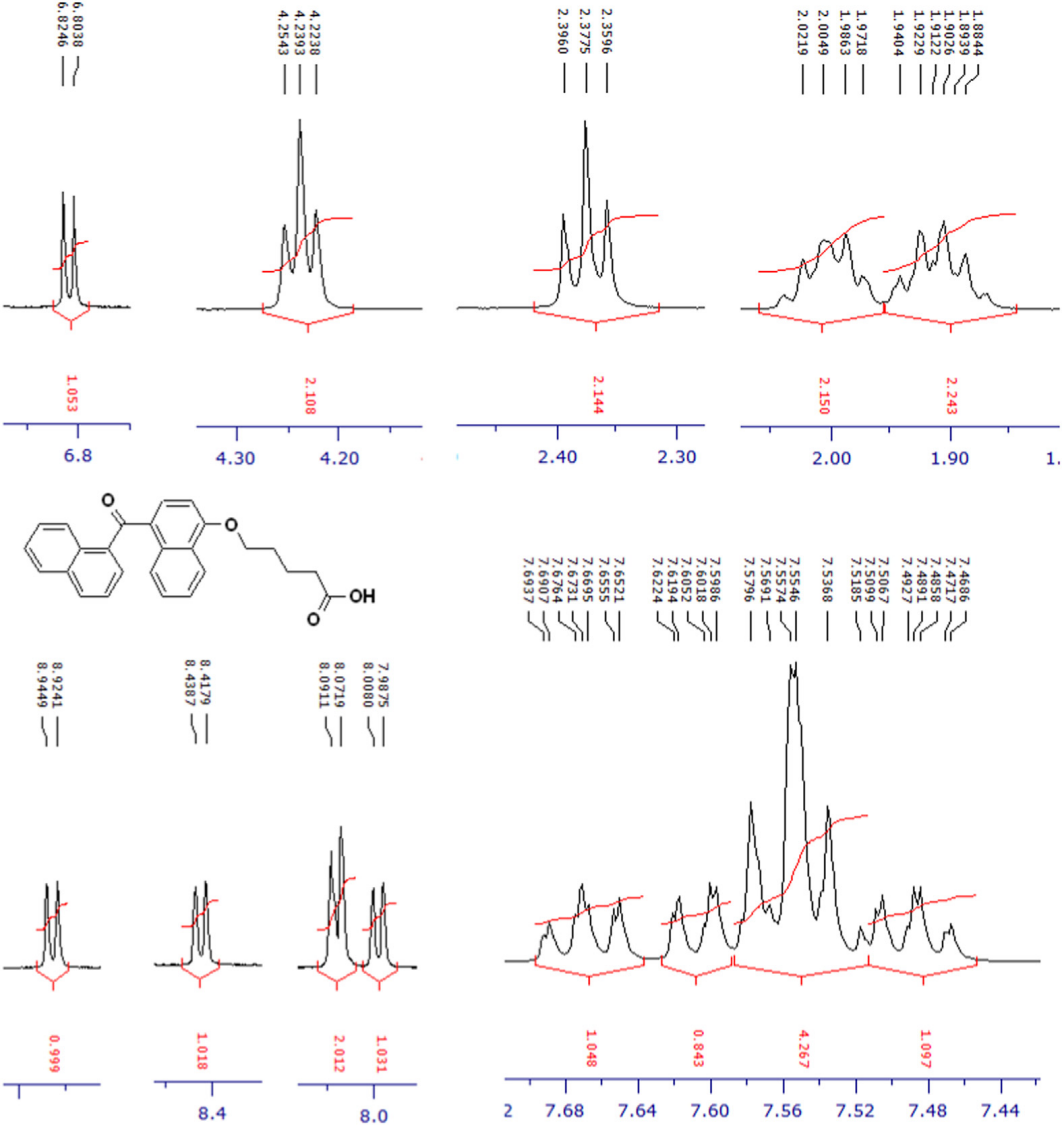
^1^H-NMR spectrum of terminally oxidized carboxylic acid metabolite of CRA13 (CD_3_OD, 400 MHz).

**Fig. 8:**
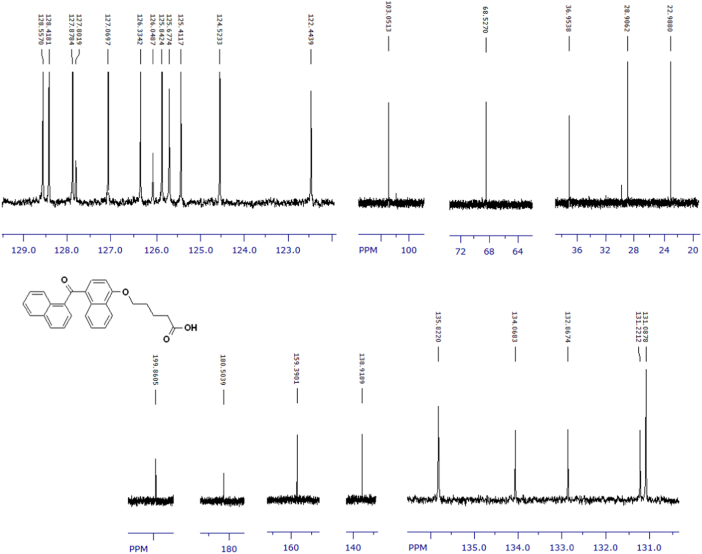
^13^C-NMR spectrum of terminally oxidized carboxylic acid metabolite of CRA13 (CD_3_OD, 125 MHz).

**Fig. 9:**
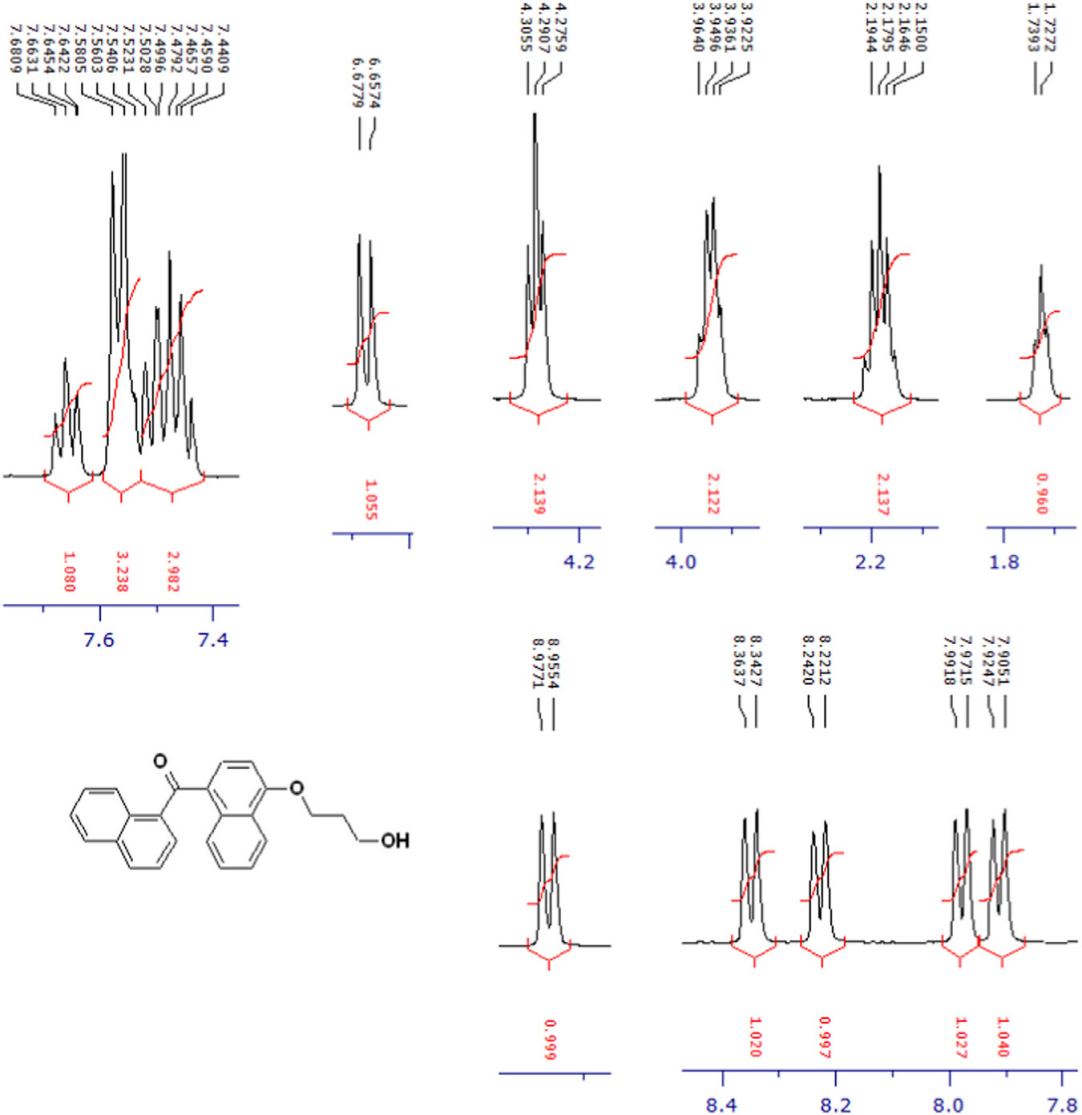
^1^H-NMR spectrum of three carbons alkyl chain analog of terminally oxidized alcoholic metabolite of CRA13 (CDCl_3_, 400 MHz).

**Fig. 10:**
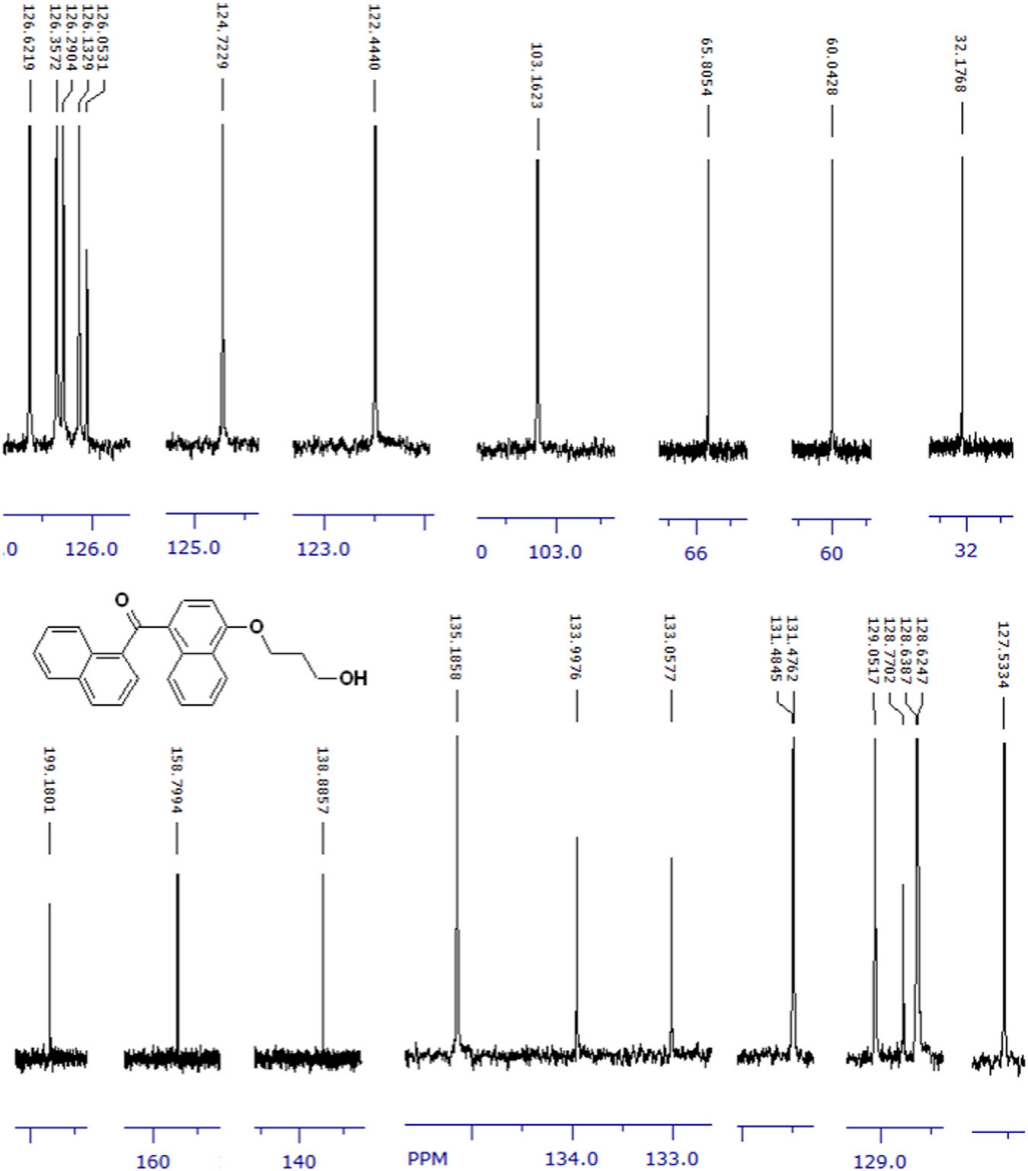
^13^C-NMR spectrum of three carbons alkyl chain analog of terminally oxidized alcoholic metabolite of CRA13 (CDCl_3_, 125 MHz).

**Fig. 11:**
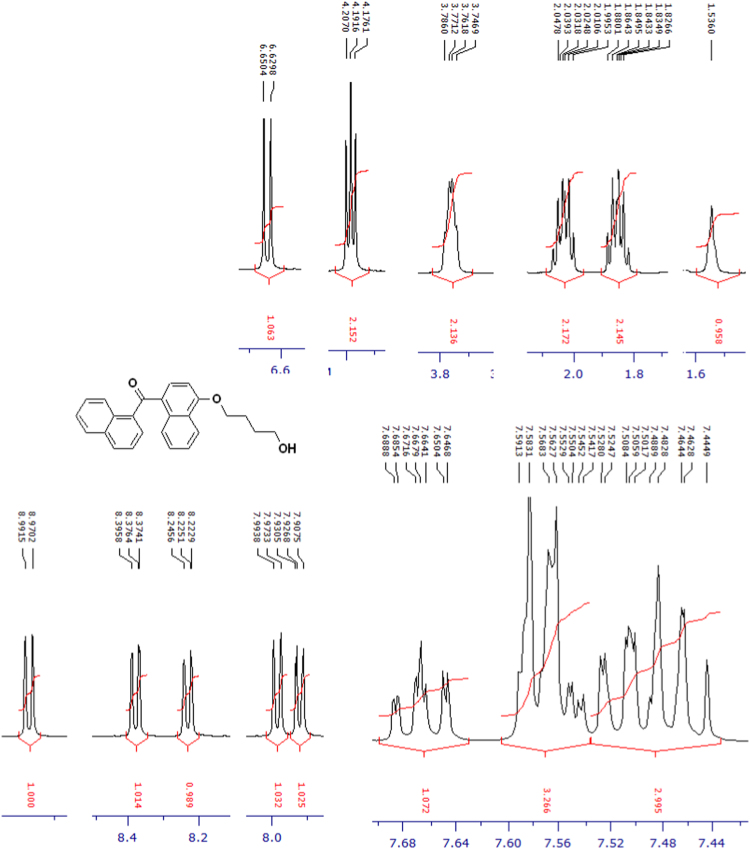
^1^H-NMR spectrum of four carbons alkyl chain analog of terminally oxidized alcoholic metabolite of CRA13 (CDCl_3_, 400 MHz).

**Fig. 12:**
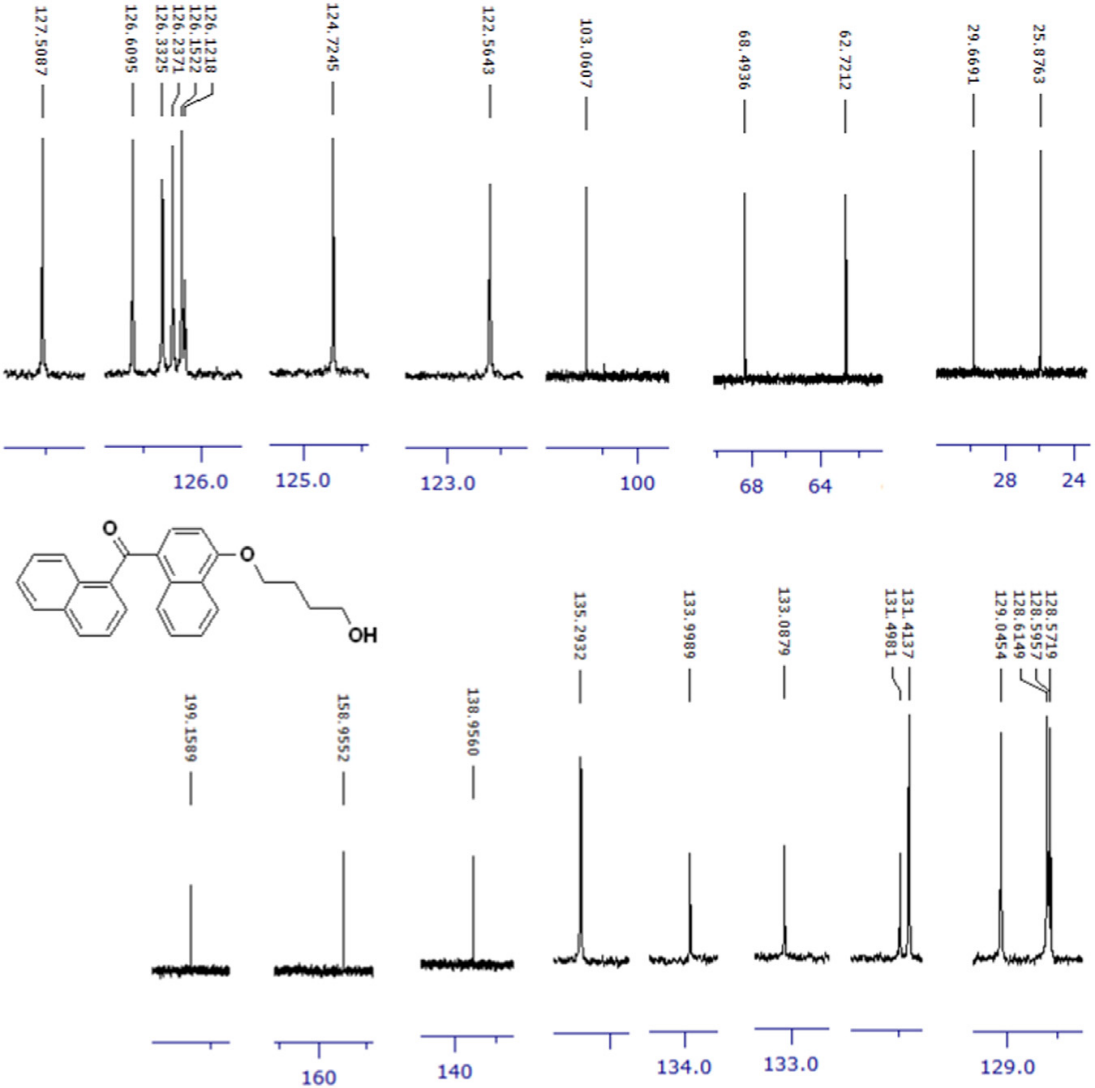
^13^C-NMR spectrum of four carbons alkyl chain analog of terminally oxidized alcoholic metabolite of CRA13 (CDCl_3_, 125 MHz).

**Fig. 13:**
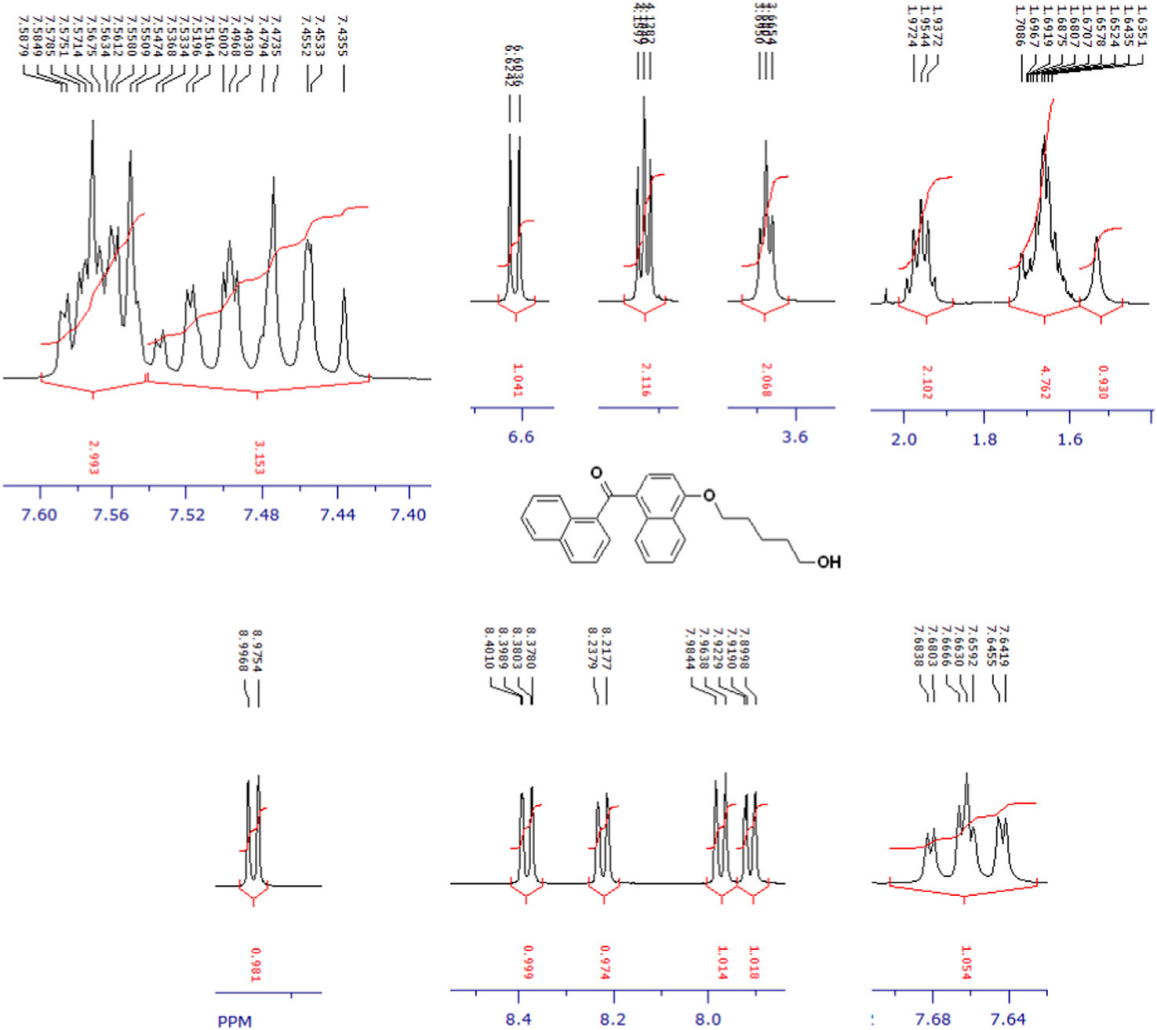
^1^H-NMR spectrum of terminally oxidized alcoholic metabolite of CRA13 (CDCl_3_, 400 MHz).

**Fig. 14:**
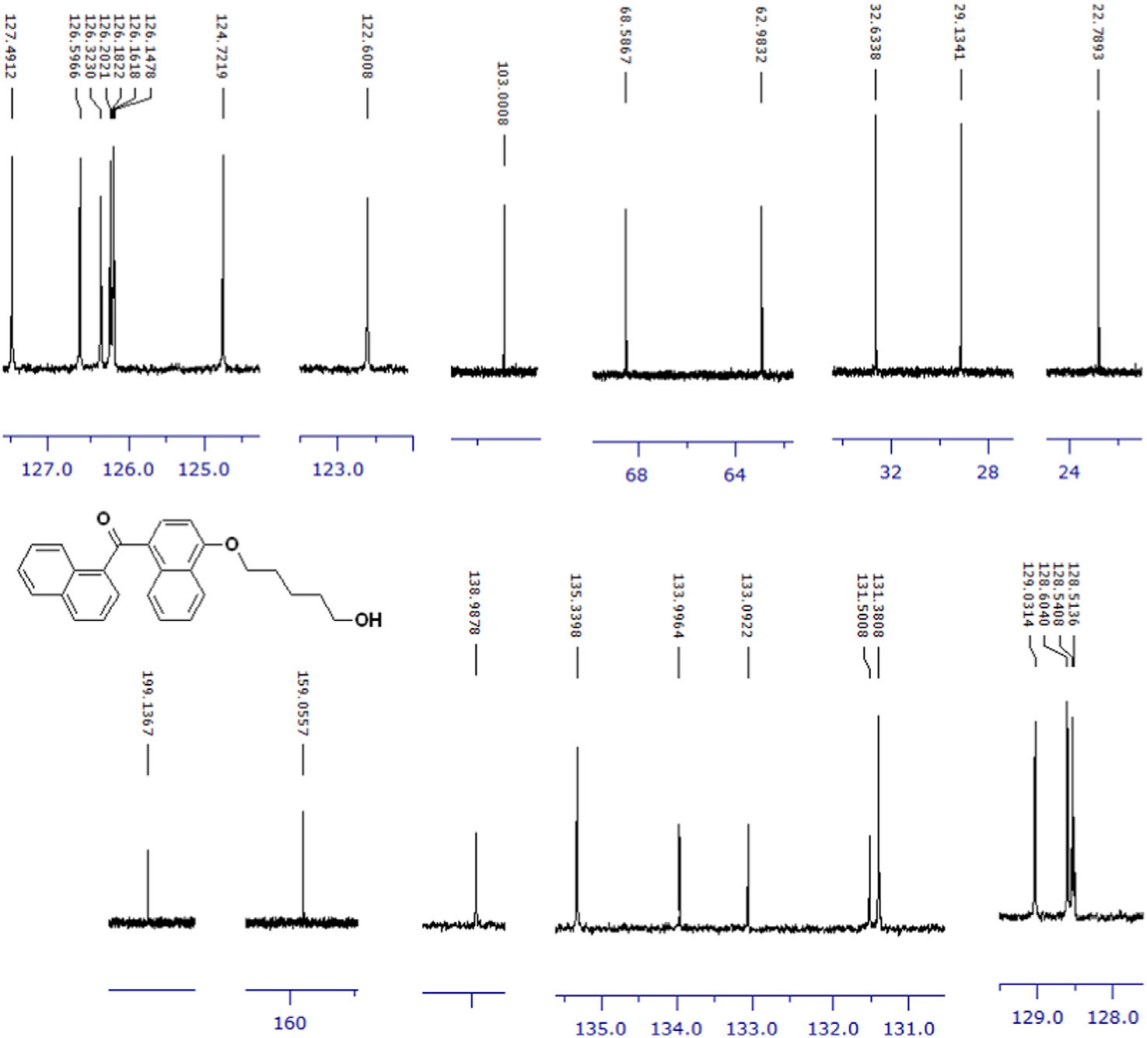
^13^C-NMR spectrum of terminally oxidized alcoholic metabolite of CRA13 (CDCl_3_, 125 MHz).

**Fig. 15:**
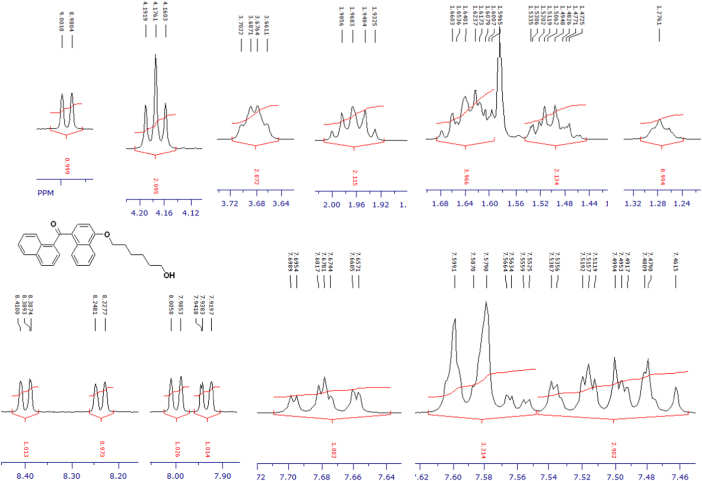
^1^H-NMR spectrum of six carbons alkyl chain analog of terminally oxidized alcoholic metabolite of CRA13 (CDCl_3_, 400 MHz).

**Fig. 16:**
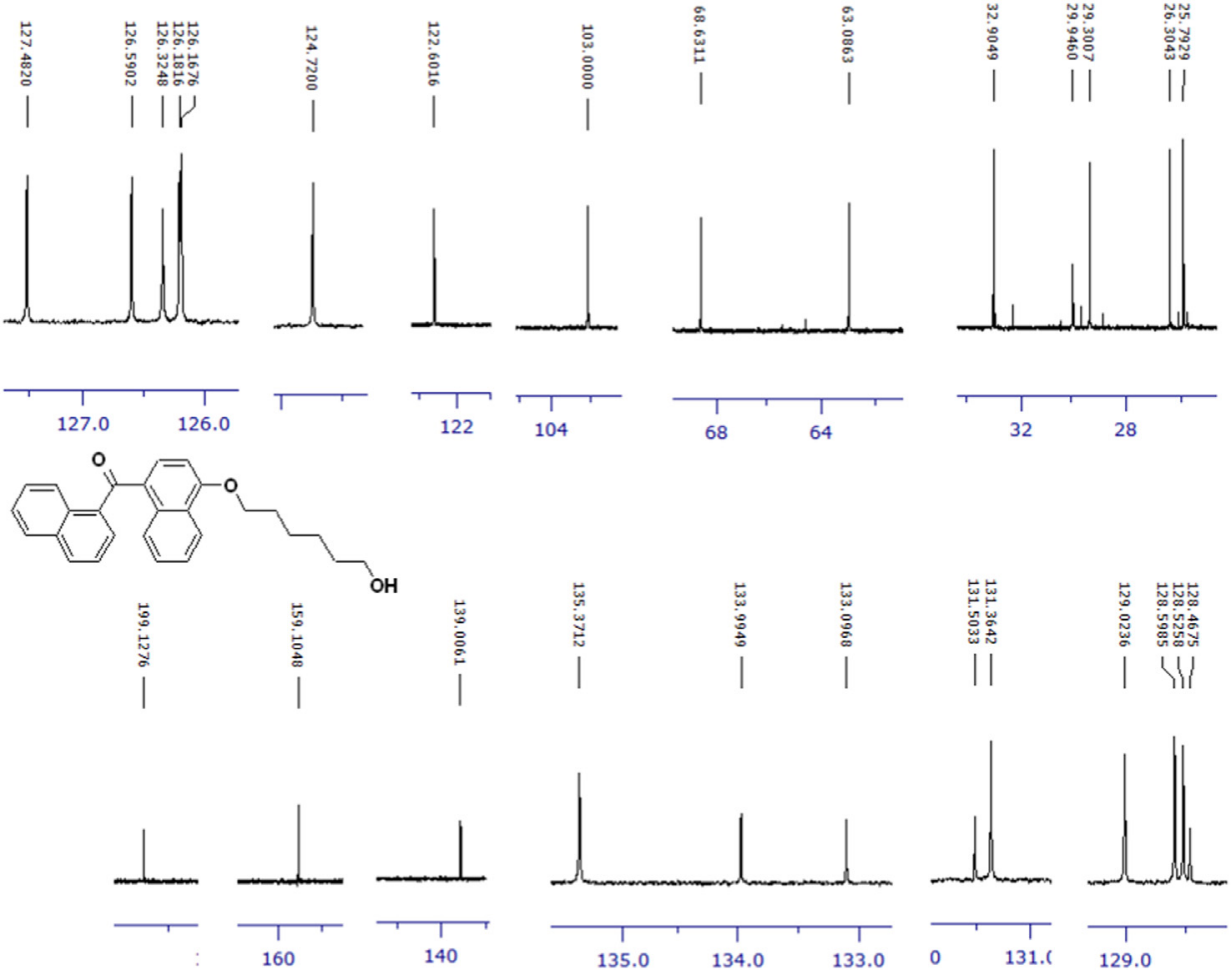
^13^C-NMR spectrum of six carbons alkyl chain analog of terminally oxidized alcoholic metabolite of CRA13 (CDCl_3_, 125 MHz).
