# Supplementary material for: 1H-NMR and 13C-NMR dataset for some oxidative metabolites of CRA13 and their analogs
Source: Data Brief. 2018 Oct 10;21:485–500. doi: 10.1016/j.dib.2018.09.069 (PMC6198130; doi:10.1016/j.dib.2018.09.069)
Supplement: Supplementary file 1 — Supplementary material [file mmc1.docx]

**CONFLICT OF INTEREST**

The Data Article I have submitted “**^1^H-NMR and ^13^C-NMR dataset for some oxidative metabolites of CRA13 and their analogs”** to the journal is original, has been written by the stated authors.

The Images that I have submitted to the journal for review are original, was taken by the stated authors, and has not been published elsewhere.

This manuscript has not been submitted to, nor is under review at, another journal or other publishing venue.

**The authors have no affiliation with any organization with a direct or indirect financial interest in the subject matter discussed in the manuscript**.
